# Clinical Equivalence of Trusynth® and Vicryl® Polyglactin 910 Sutures for Subcutaneous Tissue Closure During Cesarean Delivery: A Single-Blind Randomized Controlled Trial

**DOI:** 10.7759/cureus.39982

**Published:** 2023-06-05

**Authors:** Prema DCunha, Kranti P Silpa, Sameera M Gadwalker, Ashok K Moharana, Deepak TS

**Affiliations:** 1 Obstetrics and Gynecology, Father Muller Medical College, Mangaluru, IND; 2 Obstetrics and Gynecology, CARE Hospitals, Telangana, IND; 3 Clinical Affairs, Healthium Medtech, Bengaluru, IND

**Keywords:** tissue closure, skin disruptions, subcutaneous abdominal wound disruption, polyglactin 910 sutures, cesarean delivery

## Abstract

Background

Post-cesarean complications such as surgical site infection (SSI), bleeding, and dehiscence may occur after cesarean delivery. Subcutaneous tissue closure will reduce these complications. With this background, this study assessed the clinical equivalence of Trusynth® and Vicryl® polyglactin 910 sutures for subcutaneous tissue closure.

Methods

In this randomized, single-blind study (from January 5, 2021 to December 24, 2021), a total of 113 women with a singleton pregnancy scheduled for cesarean section were included in the study and randomized to Trusynth® (n=57) and Vicryl® (n=56) group. The primary endpoint was the incidence of subcutaneous abdominal wound disruption within six weeks of cesarean delivery. The secondary endpoints included postoperative complications (SSI, hematoma, seroma, and skin disruptions), operative time, intraoperative handling characteristics, postoperative pain, hospital stay, time taken to return to normal activities, suture removal, microbial deposits on sutures, and adverse events.

Results

No incidence of subcutaneous abdominal wound disruption was recorded. Non-significant differences in intraoperative handling parameters (except memory, p=0.007), postoperative pain, skin disruption, SSI, hematoma, seroma, hospital stay, and time to return to normal activities were observed between Trusynth® and Vicryl® groups.

Conclusion

Both Trusynth® and Vicryl® polyglactin 910 sutures can be regarded as clinically equivalent. These are safe and effective for subcutaneous tissue closure during cesarean section with minimal risk of subcutaneous abdominal wound disruptions.

## Introduction

Cesarean section reduces maternal and neonatal mortality and morbidity in an emergency situation [1], a clear benefit of which (emergency or elective cesarean) is only achieved after minimizing the postoperative risks. Cesarean sections are increasing steadily across the world [2]. India, too, has witnessed a rise from 8 to 17% (2005-2016) [3]. Post-cesarean complications, viz. surgical site infection (SSI), bleeding, and dehiscence, can complicate the mother's recovery, consequently affecting post-natal care [4]. Ideally, suturing eliminates the dead space and reduces the tension responsible for wound separation [5]. The structural properties and coating of the suture affect the healing process, failure of which leads to wound disruption and other complications, with rates ranging between 3 and 30% [6]. The risk of these complications is lowered upon subcutaneous closure [7-9]. Polyglactin 910 suture has a minimal inflammatory response and is completely absorbed by days 56-70 [10]. Though there are studies comparing polyglactin 910 with poliglecaprone-25 or nylon suture for skin closure at cesarean delivery [6,11], studies comparing two commonly used brands of polyglactin 910 suture is scarce. In this study, to demonstrate the safety and efficacy of Trusynth® polyglactin 910 suture, it has been compared with Vicryl® polyglactin 910 suture. 

## Materials and methods

Study design

This is a prospective, multicentric, two-arm, parallel-group, single-blind, randomized (1:1) controlled study conducted between January 5, 2021 and December 24, 2021. The primary objective was to compare the occurrence of subcutaneous abdominal wound disruptions within the first six weeks of cesarean delivery between Trusynth® and Vicryl® groups. The secondary objectives of this study were to compare subcutaneous wound disruptions (within 12 weeks of cesarean delivery), SSI, hematoma, seroma, tissue reaction, intraoperative suture handling parameters, time to return to normal activities, adverse events, and bacterial accumulation/growth on sutures, between the groups.

Ethical approval

The institutional ethics committees of Father Muller Medical College, Mangalore (Approval number: FMIEC/CCM/320/2020; Dated 14/07/2020) and CARE Hospitals, Hyderabad (Approval number: IEC/CARE/20539/2020/PMS; Date 19/08/2020) have approved the study. The study is registered prospectively in the Clinical Trials Registry of India (CTRI/2020/07/026824).

Participants of the study

Women aged 18-40 years with singleton pregnancy (primiparous or multiparous), requiring cesarean section (by Pfannenstiel incision), and with good systemic/mental health and surgical wound classification class I (as per CDC) were eligible to participate in the study if they agreed to provide written informed consent. Women were excluded based on hemoglobin (<7 g/dL), urogenital tract infection or chorioamnionitis within two weeks of surgery, bleeding disorders, allergy to the suture materials, stillbirth, plan for vertical skin incision, and used an experimental drug or medical device within three months of becoming pregnant. The investigators excluded participants who did not require subcutaneous suturing, provided consent to another trial, or failed to follow the study procedure or scheduled visits.

Study settings

This study was conducted at the Obstetrics and Gynecology departments of two different centers across India.

Intervention

Both Trusynth® (Healthium Medtech Limited, Bangalore, Karnataka, India) and Vicryl® (Ethicon-Johnson & Johnson, Mumbai, Maharashtra, India) are braided and coated sterile surgical sutures, indicated for soft tissue ligation and/or approximation. Both sutures are composed of 90% polyglycolic acid and 10% lactic acid.

Randomization and blinding

Computer-generated randomization was performed, and 113 subjects were randomized in a 1:1 ratio to receive either the Trusynth® (n=57) or Vicryl® (n=56) suture.

Closure of subcutaneous tissue

After screening for eligibility, the subjects underwent cesarean section at the baseline visit (Day 0). Standard care and precautions were taken pre-, peri- and post-surgery. The study sutures were used to approximate the subcutaneous adipose tissue.

Baseline characteristics

Ethnicity, age, height, weight, parity, gravida, fetal presentation, cesarean section indication, and health history were recorded for both treatment arms.

Study outcomes

Primary Endpoint

Incidence of subcutaneous abdominal wound disruptions (hematoma, seroma, or infection in the tissue between rectus fascia and skin) within six weeks of cesarean delivery was recorded.

Secondary Endpoints

Secondary endpoints included incidence of subcutaneous abdominal wound disruptions (within 12 weeks of cesarean delivery), skin disruption (spontaneous or iatrogenic separation of the wound edges of at least 1 cm in width), superficial/deep SSI [12], hematoma and seroma (collection of blood or serous fluid around the wound, respectively), suture removal, microbial deposits on sutures (in case of infection), intraoperative handling, operative time (skin incision to closure), hospital stay, time taken to return to normal activities, pain with visual analog scale (VAS), and adverse events. A five-point scale was used to rate the suture handling characteristics, viz. ease of passage through tissue, first-throw knot holding, knot tie-down smoothness, knot security, stretch capacity, memory, and suture fraying. Ratings of 1, 2, 3, 4, and 5 indicated poor, fair, good, very good, and excellent suture handling characteristics, respectively [12]. In addition, other suture-related challenges were recorded. The postoperative pain, severe, moderate, mild, and no pain, was specified by 75-100, 45-74, 5-44, and 0-4 VAS, respectively [12]. During the study period, the occurrence of any untoward medical or clinical sign, or unintended disease/injury, which was already reported as the study endpoint, was not labeled and reported as an adverse event. In addition, antibiotic prophylaxis, depth of the subcutaneous tissue, atonic postpartum hemorrhage, number and size of sutures used, perioperative complications, the outcome of surgery, and birth weight were recorded. During the study period, all the medications received by the subjects were also noted.

Sample size

In a study, after subcutaneous tissue closure with Vicryl®, a 3% wound gaping was noted after six weeks of cesarean delivery [13]. Using this assumption of a 3% event rate, 5% type I error, 80% power, and 10% non-inferiority margin, the sample size was determined as 48 in each group. Adding 20% drop-outs and post-randomization exclusion, the final sample size was calculated as 58 in each group, comprising 116 subjects in the study. After assessing eligibility per the inclusion and exclusion criteria, 57 subjects were randomized to the Trusynth® group and 56 to the Vicryl® group.

Statistical analysis

Statistical analyses were performed using SPSS (version 28.0, Chicago, Illinois, USA). Intent-to-treat (ITT) analysis set or full analysis set was used, whereby subjects in the treatment groups completed 12 weeks of follow-up. Continuous variables were compared using a t-test or Mann-Whitney U test for normally distributed and distribution-free data, respectively. Where applicable, the Chi-square test or Fisher's exact test was used to analyze qualitative variables. Statistical significance was considered as p-value <0.05. Fisher's exact test was used to analyze the study's primary endpoint. Based on the quantitative or qualitative nature of the variables, mean ± SD or proportions/percentages were used to express the secondary endpoints.

## Results

**Figure 1:**
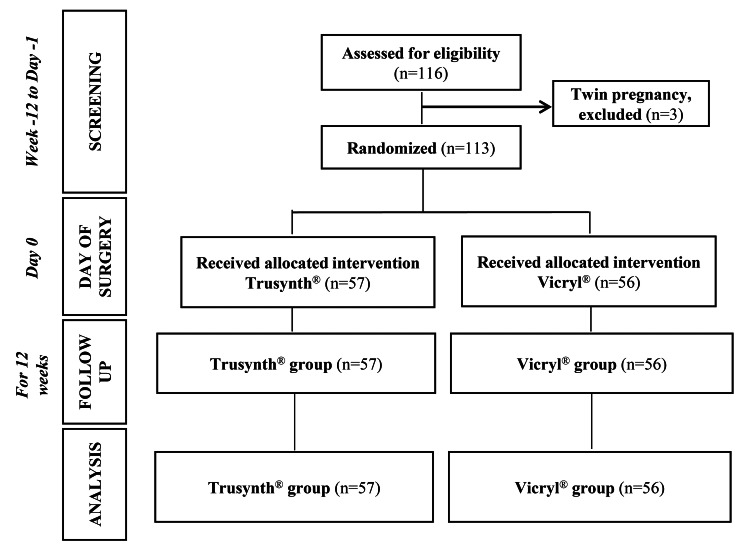
Study participants and trial design.

Between January 5, 2021 and September 26, 2021, 116 participants were screened, and a follow-up of the last subject was completed on December 24, 2021. Three subjects were screened but excluded because of having twin pregnancies. Therefore, the ITT set consisted of 113 Indian women who received the allocated intervention and appeared for all scheduled follow-ups (Figure 1).

Baseline characteristics

Baseline demographics were comparable between the groups (Table 1).

**Table 1:** Baseline demographics and obstetric details of the study participants. Continuous data are presented as Mean±SD and categorical data are presented as n (%); ^a^BMI: Body mass index.

Characteristics		Trusynth^®^ (n=57)	Vicryl^®^ (n=56)	P-value
Baseline Characteristics				
Age (years)		28.2±3.5	27.2±5.0	0.216
Weight (kg)		67.6±11.3	66.5±9.8	0.603
Height (cm)		158.0±5.3	158.5±5.2	0.601
BMI^a^ (kg/m^2^)		27.0±4.0	26.6±4.2	0.545
Obstetric Details				
Gestation period (weeks)		37.6±2.4	37.5±2.3	0.753
Parity number	0	36 (63.2%)	36 (64.3%)	0.762
	1	16 (28.1%)	17 (30.4%)	
	2	4 (7.0%)	3 (5.4%)	
	≥3	1 (1.8%)	0	
Gravida	1	24 (42.1%)	29 (51.8%)	0.272
	2	20 (35.1%)	12 (21.4%)	
	≥3	13 (22.8%)	15 (26.8%)	
Fetal presentation	Breech	2 (3.5%)	1 (1.8%)	0.569
	Vertex	55 (96.5%)	55 (98.2%)	
Indication for cesarean section	Fetal distress	35 (61.4%)	38 (67.9%)	0.646
	Intrauterine growth restriction	4 (7.0%)	3 (5.4%)	
	Gestational diabetes mellitus	3 (5.3%)	1 (1.8%)	
	Gestational hypertension	2 (3.5%)	0	
	Previous LSCS	13 (22.8%)	13 (23.2%)	
	Transverse arrest	0	1 (1.8%)	

In Trusynth® and Vicryl® groups, 25 (43.9%) and 21 (37.5%) subjects, respectively, had medical/surgical history (p=0.567). Birth weight of the infant showed no significant difference between Trusynth® and Vicryl® groups (2.8±0.6 vs. 2.7±0.7 Kg, p=0.255).

Primary endpoint analysis

There was no incidence of subcutaneous abdominal wound disruptions during the entire study period in both the groups.

Secondary endpoint analysis

Intraoperative Profile

Antibiotic prophylaxis, as per institutional practice, was given to all subjects. In all subjects, the interrupted suturing technique was used for subcutaneous tissue closure using one suture of size 1. Incidences of atonic postpartum hemorrhage and other perioperative complications were not recorded in any group. Other intraoperative characteristics are given in Table 2.

**Table 2:** Intraoperative and postoperative profiles of the study participants. Continuous data are presented as Mean ± SD and categorical data are presented as n (%).

Characteristics		Trusynth^® ^(n=57)	Vicryl^® ^(n=56)	P-value
Intraoperative Profile				
Total operative time (minutes)		73.0 ± 22.8	74.5 ± 25.7	0.735
Thickness of subcutaneous tissue		2.2 ± 1.1	2.1 ± 1.3	0.824
Thickness of subcutaneous tissue	0-2 cm	26 (45.6%)	26 (46.4%)	0.927
	2.1-6 cm	31 (54.4%)	30 (53.6%)	
Post-operative Profile				
Time of onset of pain after surgery (hours)		3.4 ± 2.3	3.6 ± 3.0	0.865
Number of analgesics prescribed	Day 0	1.4 ± 0.7	1.6 ± 0.8	0.361
	Day 3	1.3 ± 0.6	1.3 ± 0.6	0.586
	Day 4-7	1.0 ± 0.3	1.0 ± 0.3	0.991
	Week 6	0.02 ± 0.1	0	0.324
	Week 12	0	0.02 ± 0.1	0.315
Hospital stay (days)		3.7 ± 1.9	3.7 ± 1.0	0.845
Return to normal day-to-day activity (days)		19.4 ± 6.4	20.2 ± 7.3	0.495

**Figure 2:**
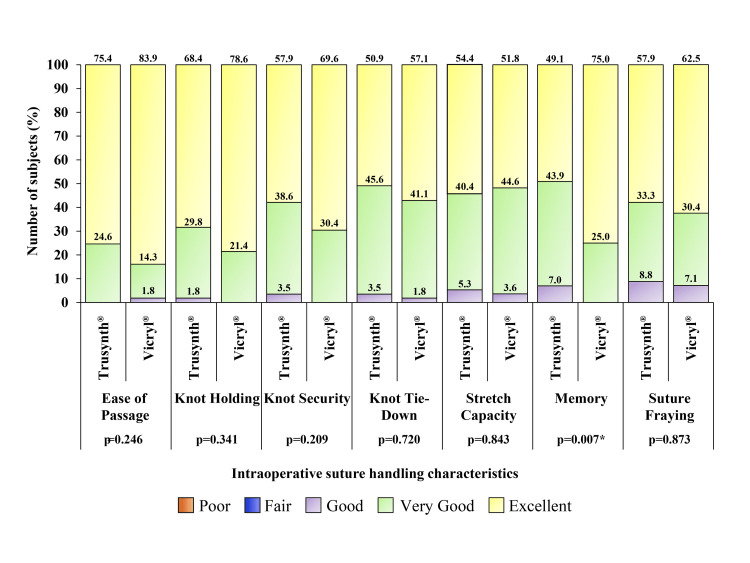
Intraoperative suture handling characteristics of the study participants assigned to Trusynth® (n=57) and Vicryl® (n=56) group Percentage scores for “Excellent”, “Very good” and “Good” is shown in one bar for different suture handling characteristics. None of the characteristics had fair or poor score. *p<0.05

The result of suture handling characteristics, viz., ease of passage, knot holding, security, tie-down, stretch capacity, and suture fraying, were comparable between the groups (Figure 2). 

A significant difference (p=0.007) regarding memory was observed between the groups. "Very good" scores for memory were high in the Trusynth® group, whereas "Excellent" scores were high in the Vicryl® group. No suture-related challenges were noted in the groups. Both groups showed good outcomes of surgery.

Postoperative Profile

No incidence of skin disruption, hematoma, seroma, and superficial/deep SSI was noted in any subject. Neither suture was extruded nor removed and sent for culture for both groups. During week 6, one (1.8%) subject in the Trusynth® group was readmitted to the hospital due to a urinary tract infection, which was reported as a serious adverse event. The result showed no significant difference (p=0.319) between the groups. Other postoperative characteristics are presented in Table 2.

**Figure 3:**
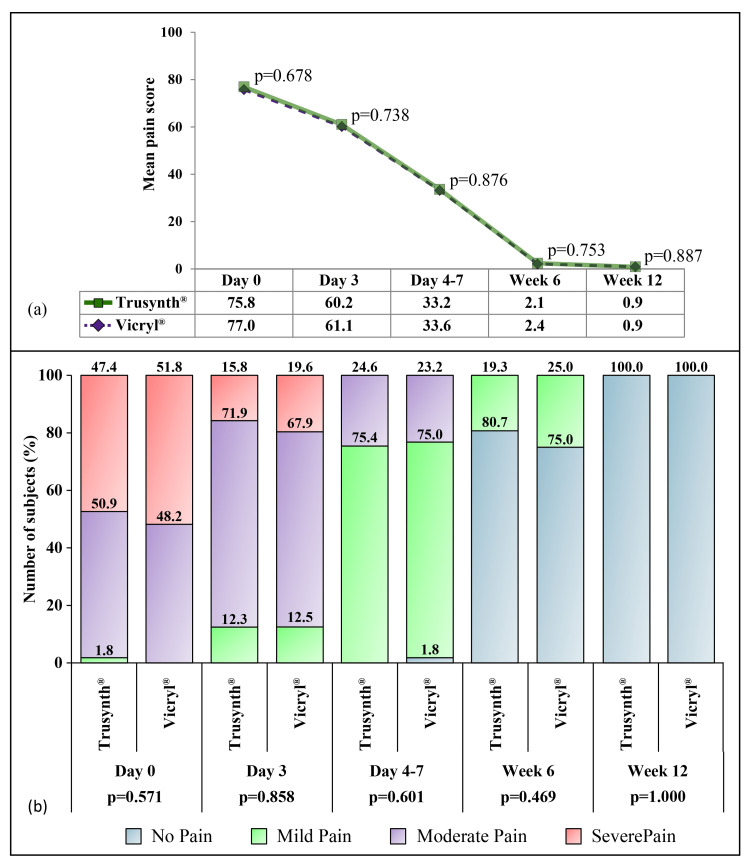
Change in pain across the follow-up visits in study participants assigned to Trusynth® (n=57) and Vicryl® (n=56) groups. (a) Mean pain score was evaluated using the VAS scale, (b) Frequency of subjects with different grades of pain (mild, moderate, and severe). VAS: Visual analog scale.

After the surgery, the mean pain VAS scores were 75.8 and 77.0 for Trusynth® and Vicryl® groups, respectively, gradually decreasing in subsequent visits to become 0.9 in both groups by week 12. The results depicted non-significant differences in pain between the groups (Figures 3a and 3b).

In the Trusynth® group, 1 (1.8%) subject had a fever, 1 (1.8%) subject had hypertension, and 1 (1.8%) subject had general body pains. In the Vicryl® group, fever in 1 (1.8%) subject and spinal headache in 1 (1.8%) subject were recorded. At week 6, a serious adverse event (hospitalization due to urinary tract infection) was noted in 1 (1.8%) subject of the Trusynth® group. The subject continued the study after recovery. Both the adverse and severe adverse events were not suture-related. The concomitant/prescribed medications taken by the majority of the subjects during the study period are shown in Table 3.

**Table 3:** Concomitant or prescribed medications in the study participants during the study period.

Prescribed Medications	Trusynth^®^ (n=57) n (%)	Vicryl^®^ (n=56) n (%)
Analgesics		
Paracetamol	46 (80.7)	45 (80.4)
Tramadol	25 (43.9)	26 (46.4)
Butorphanol	18 (31.6)	22 (39.3)
Diclofenac	17 (29.8)	17 (30.4)
Paracetamol + Aceclofenac	20 (35.1)	19 (33.9)
Antibiotics		
Cefuroxime	29 (50.9)	28 (50.0)
Metronidazole	31 (54.4)	29 (51.8)
Ceftriaxone	27 (47.4)	26 (46.4)
Cefpodoxime	16 (28.1)	11 (19.6)
Cefuroxime + Clavulanic acid	11 (19.3)	10 (17.9)

## Discussion

The global increase in cesarean delivery by 4.4% yearly [14], along with 28.1% and 12.0% rise in private and public sector health facilities in India [15], manifest the requirement for evidence-based approaches to minimize post-cesarean complications and improve surgery outcomes. Subcutaneous tissue closure with suturing, especially for wound thickness of >2 cm, is proven to be effective in the reduction of wound complications [16]. Previous studies compared polyglactin 910 with triclosan-coated polyglactin 910 [17], nylon [11], plain catgut [18], and polypropylene sutures [19] in cesarean section. This study compared the two commonly used polyglactin 910 sutures brands, Trusynth® and Vicryl®. Polyglactin 910 suture has good handling properties, reduced tissue drag, and good knot security [10]. Similarly, the current findings depicted "Good," "Very good," and "Excellent" scores for individual aspects of handling for both suture brands. If wound healing is not complete, it leads to wound disruption, i.e., partial or complete separation of the approximated tissue [20]. Around 4-29% of abdominal wound disruptions are reported in all Obstetrics and Gynaecological surgeries [21]. Post-cesarean wound complications were reported in 5.3% [22] and 2.3% of women [23]. Suture closure of subcutaneous tissue lowered the seroma rate, not hematoma or wound infections [16]. Another study reported decreased incidence of postoperative hematoma and no incidence of wound disruption, SSI, and seroma after subcutaneous tissue closure [24]. In the present study, post-cesarean subcutaneous tissue closure with polyglactin 910 sutures resulted in no incidence of subcutaneous abdominal wound disruptions, superficial/deep SSI, skin disruption, hematoma, and seroma, indicated efficacy and bacterial resistance of both Trusynth® and Vicryl® sutures. Post-surgery pain was reported to be comparatively lower after skin closure with an absorbable suture following subcutaneous fascia closure [4]. Another study demonstrated that 78% of women were not using pain medication by 4-8 weeks postpartum [25]. Even in this study, who had pain after subcutaneous closure, showed improvement with each follow-up visit along with a decreasing requirement of analgesics with each follow-up (~0 by week 6), implicating recovery of the subjects. The time taken to return to normal day-to-day activity was also similar between the groups. Regarding safety, no device-related adverse events were recorded in both arm. Although a serious adverse event was observed in the study, that, too, was not related to the study device. The limitation of the present study includes a potential bias to favor one suture over the other, as the study's investigators were not blinded.

## Conclusions

There were non-significant differences in the proportion of subjects having an incidence of subcutaneous abdominal wound disruptions, skin disruption, SSI, hematoma, and seroma. The total operative time, hospital stay duration, intraoperative suture handling (except memory), return to normal day-to-day activities, pain, and adverse events were also not significantly different between the two groups. The results indicated clinical equivalence of Trusynth® and Vicryl® suture. Therefore, both the Trusynth® and Vicryl® polyglactin 910 sutures can be safely and effectively used for subcutaneous tissue closure during cesarean section with minimal risk for subcutaneous abdominal wound disruptions.
